# Membrane ATPases and Mitochondrial Proteins in Fetal Cerebellum After Exposure to L-Glutamate During Gestation

**DOI:** 10.3390/membranes15050152

**Published:** 2025-05-16

**Authors:** Adrián Tejero, David Agustín León-Navarro, Mairena Martín

**Affiliations:** Department of Inorganic and Organic Chemistry and Biochemistry, Faculty of Chemical Sciences and Technologies, Institute of Biomedicine, Investigación Sanitaria de Castilla-La Mancha, University of Castilla-La Mancha, Avenida Camilo José Cela 10, 13071 Ciudad Real, Spain; adrian.tejero@uclm.es (A.T.); mairena.martin@uclm.es (M.M.)

**Keywords:** L-glutamate, Mg^2+^-ATPase, mitochondria

## Abstract

L-Glutamate (L-Glu) and its salt derivatives are widely used in the food industry as flavor enhancers. Although the consumption of these compounds is generally considered safe, some studies suggest that chronically consuming L-Glu may be associated with various disorders. In this study, Wistar pregnant rats were treated daily with 1 g/L of L-Glu in their drinking water throughout the gestational period. OPA-1, DRP-1, and mitofusin 2—key proteins involved in mitochondrial fusion and fission—were analyzed by Western blot. The results showed that L-Glu exposure significantly decreased DRP-1 levels, while OPA-1 and mitofusin 2 levels were unaffected. This was accompanied by a notable decrease in mitochondrial complexes III and V. The activities of Mg^2+^-ATPase and Na^+^/K^+^-ATPase were also analyzed in fetal cerebellar plasma membranes. Maternal L-Glu intake significantly increased Mg^2+^-ATPase activity. Regarding Na^+^/K^+^-ATPase, the data showed that L-Glu exposure did not modulate the protein level or its activity. However, a positive interaction with glutamate receptors was observed in both activities, although neither AMPA nor NMDA receptors appeared to be involved. These results suggest that chronic maternal L-Glu intake during gestation modulates Mg^2+^-ATPase activity and protein markers of mitochondrial dynamics in the fetal cerebellum, which could affect neonatal development.

## 1. Introduction

Under physiological conditions, endogenous glutamate participates in multiple functions, such as energy production in enterocytes [1], which is a precursor of the powerful antioxidant glutathione [2], and as an excitatory neurotransmitter in the nervous system [3]. It is not surprising, therefore, that chronic L-Glu intake has been associated with a wide variety of disorders, including obesity, metabolic issues, alterations in the reproductive system, and neurotoxic effects [4,5]. Glutamate intake from the diet varies across different countries and cultures, but it may pose a risk when taken in excessive consumption [6]

L-Glutamate (L-Glu) and its salt derivatives, such as monosodium glutamate (MSG), are widely used by the food industry due to their ability to induce a unique taste called umami and enhance the palatability of foods [7,8]. These molecules are present in a wide variety of processed foods, including frozen meals, fast foods, soups, and canned tuna [9]. It is estimated that the average daily human intake in European countries ranges from 0.3 to 1 g [10]. The ability of exogenous glutamate to modulate brain behavior, neurochemistry, and even neuromorphology is currently being confirmed using different models [6].

Our research group has recently published that chronic maternal L-Glu intake during gestation evokes oxidative stress in the fetal cerebellum [11]. MSG-induced oxidative stress has also been reported in rat striatum and cerebellum [12,13]. The cerebellum plays a crucial role in maintaining motor coordination, sensory perception, and controlling voluntary movement. It is also involved in motor learning and is highly vulnerable to injury. Consequently, damage to this region can disrupt learning processes, leading to motor impairments or ataxia [14].

Oxidative stress is a condition characterized by an imbalance between the production and accumulation of reactive oxygen species (ROS) and the antioxidant defenses [15]. In addition to their central role in ATP production, mitochondria are also the primary source of ROS in the cell. It is estimated that during intense oxidative phosphorylation, between 1 and 2% of the oxygen consumed by the cell is converted into ROS. This generation of free radicals, such as superoxide anion, is primarily driven by the flow of electrons through the mitochondrial electron transport chain [16].

Although under physiological conditions, the production of ROS in mitochondria is necessary, as they participate in crucial processes, such as immune defense against pathogens and cell signaling [17], the presence of oxidative stress is a clear symptom of mitochondrial alteration. Mitochondria are highly dynamic organelles that undergo coordinated cycles of fusion and fission processes. During fusion, several mitochondria can merge into new mitochondria, a process in which the protein OPA-1 participates. On the other hand, mitochondrial fission allows mitochondrions to fragment into several smaller ones. The protein Drp-1 plays a key role in this process. An adequate balance between fusion and fission is necessary for many cellular processes such as the cell cycle, immunity, and apoptosis. However, it has been observed that CNS insults can promote an imbalance, favoring mitochondrial fission [18,19,20]. Currently, it remains unknown whether the chronic oral intake of L-Glu can alter normal mitochondrial dynamics.

Progressive free radical generation and reduced antioxidant levels have been linked to impaired ATPase function [21]. Evidence shows that ATPases like Ca^2+^-ATPase, Mg^2+^-ATPase, and Na^+^/K^+^-ATPase are crucial for maintaining ionic gradients and nerve cell functions, including signal transduction, neurotransmitter release, synaptic plasticity, and cognitive processes in the CNS [22,23], and even influencing glutamate signaling in neurodegenerative diseases [24].

Accordingly, the activity of Na^+^/K^+^-ATPase is crucial for maintaining proper excitability in the nervous system [17], and increased ROS levels modify its subunits, leading to the loss of activity and even the degradation of the protein [18]. Moreover, this pump can activate signaling pathways that result in the phosphorylation of proteins involved in apoptosis and cellular metabolism [25].

Several proteins and receptors can bind to and impair its activity, and even some studies have demonstrated a co-localization and physical interaction between Na^+^/K^+^ ATPase subunits and glutamate transporters [26] and receptors, [27] including ionotropic glutamate receptors such as NMDA [28] for which some findings reveal a potential cross-talk between this receptor and the pump, playing important roles in the regulation of learning and memory and AMPA, which colocalizes and associates with Na^+^/K^+^ ATPase during synaptic transmission [29]; this is critical for the formation of synaptic plasticity such as long-term potentiation and depression (LTP and LTD). The inhibition of this activity in the cerebellum may potentiate glutamate excitotoxicity, evoking cytotoxic effects such as Ca^2+^ influx. Previous studies have shown that glutamate can increase cerebellar Na^+^/K^+^ ATPase activity through the cyclic GMP-PKG pathway [30] via tissue preincubation with L-Glu [31]. However, to our knowledge, there are no data analyzing the consequences of the chronic oral consumption of L-Glu (not MSG) on the activity of this Na^+^/K^+^-ATPase.

Mg^2+^-ATPase is another essential ion transporter involved in maintaining a high intracellular concentration of magnesium ions. These ions play a key role in the synthesis and metabolism of lipids, carbohydrates, proteins, and nucleic acids. Additionally, it participates in the synthesis of the antioxidant defense system [32]. However, the impact of oxidative stress on Mg^2+^-ATPase activity has been less studied. Some authors have reported that oxidative stress induces an increase in the activity of this ionic pump [33,34], while others have observed a significant decrease [35,36].

Therefore, the aims of the present study were to analyze whether maternal chronic oral L-Glu treatment during gestation could alter the levels of protein markers related to mitochondrial function and dynamics and whether these changes could be associated with modifications in the activities of both Na^+^/K^+^-ATPase and Mg^2+^-ATPase in the cerebellum.

## 2. Materials and Methods

### 2.1. Animal Treatment

In the present work, pregnant Wistar rats were kept on a 12 h light/12 h dark cycle, with light appearing at 7:00 am and free access to food and drinking water. Pregnant rats were supplemented with a dose of 1 g/L of L-Glutamate in their drinking water from gestation day 2 onwards during the entire gestation period. At the end of this period, pregnant rats were sacrificed, and the fetuses were delivered surgically. Fetal cerebellums were then frozen in liquid nitrogen and stored at −70 °C. The mean amount of glutamate consumption for the L-Glu-treated group was 110 ± 4.6 mg/Kg·day, and daily water consumption was significantly lower in the L-glu-treated group, with no effect on food intake or body weight [37]. As the average daily human consumption of glutamate in Asian and European countries ranges from 2.2 to 12 g approximately [10,38] and the average weight is about 75 Kg, we can assume that an approximation of the human consumption of L-Glu is 30 to 160 mg/Kg·day. Therefore, the average values obtained for the consumption of this amino acid in gestational rats can represent those values usually consumed in humans. All experiments followed the European Community regulations regarding animal experimentation and those of the Animal Experimentation Committee of Castilla-La Mancha University (registration number 1103.01).

### 2.2. Plasma Membrane Isolation

Cerebellar plasma membranes were isolated according to the protocol previously described [39]. In brief, the fetal cerebellums were homogenized in 20 volumes of an isolation buffer (50 mM Tris-HCl, 10 mM MgCl_2_, pH 7.4) with protease inhibitors, using a Dounce homogenizer with pestle A (10 strokes) followed by pestle B (10 strokes). The homogenate was then centrifuged at 1000× *g* for 5 min in a Beckman JA 21 centrifuge (Coulter, Madrid, Spain). The resulting supernatant was further centrifuged at 27,000× *g* for 10 min to separate the plasma membrane (pellet) and cytosolic fractions. The pellet was resuspended in an isolation buffer, and protein concentration was determined using the Lowry method with bovine serum albumin (BSA) as the standard.

### 2.3. Immunoblotting Assay

Homogenates and plasma membrane samples (30 μg) were subjected to 10–15% polyacrylamide gel electrophoresis and sodium dodecyl sulfate. Proteins were transferred to nitrocellulose membranes using the iBlot™ Dry Blotting System (Invitrogen, Barcelona, Spain) and blocked for 60 min with 5% non-fat skimmed milk in phosphate-buffered saline. Then, immunodetection was performed by incubating the nitrocellulose membranes with the following subsequent antibodies: the Anti-OPA1 (1:1000, ab42364 from Abcam. Cambridge, United Kingdom), Anti-DRP1 antibody (1:1000, ab184247 from Abcam), Anti-mitofusin 2 antibody (1:1000, ab124773 from Abcam), OXPHOS antibody (1:1000, ab110413 from Abcam). β-Actin antibody (1:5000, ab8226 from Abcam), and anti-β-Tubulin, clone AA2 (1:5000, 05-661 from Millipore), were used as protein loading controls. After washing, blots were incubated with horseradish peroxidase coupled with goat anti-mouse or anti-rabbit IgG (GAMPO 170–6516 and 1:4000 and GARPO 172–1019 1:4000 from Bio-Rad, Kai Tak, Hong Kong). Bands were visualized using the ECL chemiluminescence detection kit from GE Healthcare (Madrid, Spain) in a G: Box chamber and luminescent bands were quantified by densitometry using Gene-Tools software version 4.01 (Syngene). The results are presented in arbitrary units (the ratio between the protein of interest and β-actin).

### 2.4. Na^+^/K^+^-ATPase and Mg^2+^-ATPase Activities Assay

Plasma membrane fractions (1 mg/mL) were used for this procedure. Briefly, the reaction buffer contained 5.0 mM of MgCl_2_, 80.0 mM of NaCl, 20.0 mM of KCl, and 40.0 mM of Tris–HCl, pH 7.4, in a final volume of 200 μL. The addition of 3 mM freshly prepared ATP initiated the reaction. The Mg^2+^-ATPase activity was measured after the addition of 1.0 mM of ouabain. Then, the Na^+^/K^+^-ATPase activity was measured as the difference between the total ATPase activity and the Mg^2+^-ATPase activity. In our experimental conditions, only 6% and 8% of this activity corresponded to ATP synthase and SERCA, respectively. The inorganic phosphate released was measured based on the spectrophotometric quantification of the phosphomolybdate–malachite green complex as described by Chan et al. 1986 [40]. The results are expressed as the nmol of Pi released/min·mg of protein. The effect of different glutamate receptor ligands such as AMPA, NMDA, and glutamate on these enzymatic activities was measured following the same procedure, but with a 6 min preincubation time with 140 μM AMPA, NMDA, and L-Glutamate each at 30 °C with stirring. The results were expressed as the percentage of the action of each ligand with respect to the basal activity of Na^+^/K^+^ and Mg^2+^-ATPase, respectively.

### 2.5. Statistical and Data Analysis

Results are expressed as the mean ± Standard Error of the Mean (SEM). Statistical comparisons were performed using unpaired two-tailed Student’s *t*-test and a two-way ANOVA, followed by a Bonferroni comparison post hoc test using the GraphPad Prism 8.0 program (GraphPad Software, San Diego, CA, USA). The differences between mean values were considered statistically significant at * *p* < 0.05.

## 3. Results

### 3.1. Maternal Chronic L-Glu Intake During Gestation Alters the Mitochondrial Oxidative Phosphorylation Complex III and V in Fetal Cerebellum

**Figure 1:**
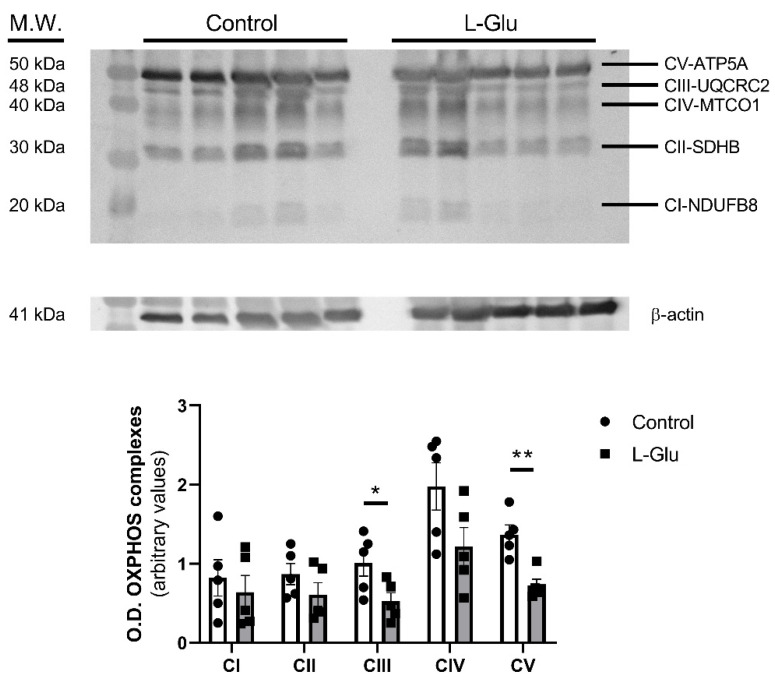
Western blot of OXPHOS in fetal cerebellum exposed to L-Glutamate during gestation. Histograms show data that correspond to five different complexes, represented as mean ± SEM of 5 different experiments performed using different tissue homogenates. * *p* < 0.05 or ** *p* < 0.01, significantly different from control values according to Student’s *t*-test.

First, we aimed to determine whether chronic L-Glu intake during gestation could affect mitochondrial oxidative function by modulating mitochondrial complexes. To this end, the expression of mitochondrial complexes was analyzed in cerebellar homogenates from fetuses exposed to maternal L-Glu intake and compared with the corresponding control and L-Glu-treated groups. As shown in Figure 1, maternal L-Glu intake significantly decreased the level of mitochondrial complexes III (1.010 ± 0.166% vs. 0.526 ± 0.107 arbitrary units, *p* < 0.05) and V (1.370 ± 0.121% vs. 0.726 ± 0.078 arbitrary units, *p* < 0.01) while the other complexes (I, II and IV) were not significantly altered.

### 3.2. Maternal Chronic L-Glu Intake During Gestation Alters the Levels of the Proteins Involved in the Dynamics of the Mitochondria in Fetal Cerebellum

**Figure 2:**
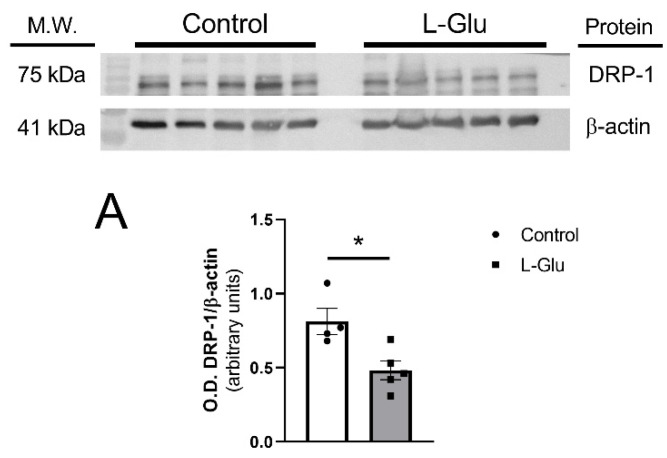

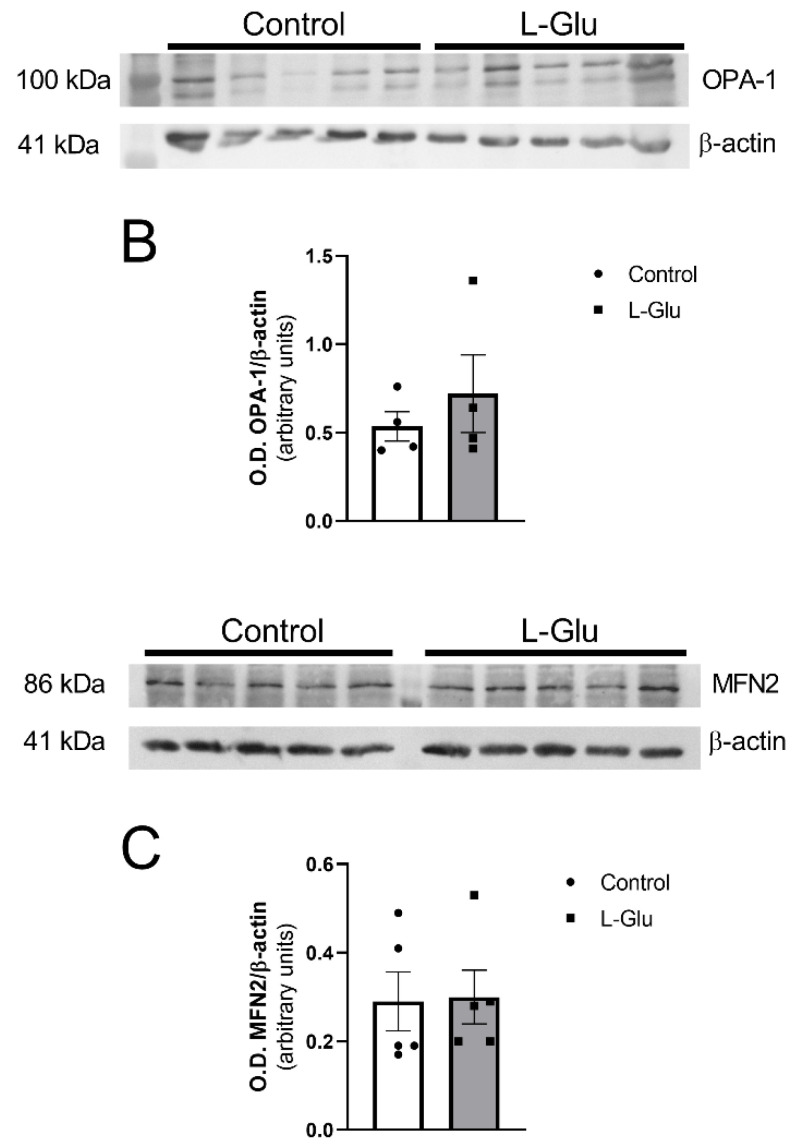
Western blot and densitometric analyses of the corresponding bands of mitochondrial dynamic markers DRP-1 (**A**), OPA-1 (**B**), and mitofusin 2 (MFN2) (**C**) in fetal cerebellum exposed to L-Glutamate during gestation. Histograms show data that are represented as mean ± SEM of 5 different experiments performed using different tissue homogenates. * *p* < 0.05, significantly different from control values according to Student’s *t*-test.

Next, we sought to determine whether the maternal oral consumption of L-Glu during gestation could also modulate mitochondrial dynamics in the fetal cerebellum. To investigate this, we analyzed the levels of three key proteins, OPA-1, DRP-1, and mitofusin 2, as they play crucial roles in mitochondrial fission (DRP-1) and fusion (OPA-1 and mitofusin 2). As shown in Figure 2, DRP-1 levels were significantly reduced in the cerebellum of fetuses exposed to maternal L-Glu intake (0.813 ± 0.088 vs. 0.482 ± 0.063 arbitrary units, *p* < 0.05). No significant variations were observed in OPA-1 and mitofusin 2 protein levels.

This oxidative stress induced by chronic maternal L-Glutamate intake could also be seen as altering ATPase activity. Indeed, ATPases are the primary consumers of energy in the cell, and several studies have reported that ATP deficiency and oxidative stress may impair Na^+^/K^+^-ATPase activity, contributing to neuronal death [41]. Moreover, Mg^2+^-ATPase could also be affected by oxidative stress, as magnesium ions play an important role in the brain by counteracting oxidative damage, although the exact mechanisms for this remain unclear [42,43]. For this reason, we decided to investigate the effect of maternal L-Glutamate intake on ATPase activities.

### 3.3. Maternal Chronic L-Glu Intake During Gestation Alters the Activity of Mg^2+^-ATPase in Fetal Cerebellum

As indicated in the previous paragraph, the next point we investigated was the effect of maternal L-Glu consumption on Mg^2+^-ATPase activity. We addressed the following three questions:

(1) Is Mg^2+^-ATPase activity affected by maternal L-Glu consumption during gestation?
(2) Can the activation of glutamate receptors modulate the activity of Mg^2+^-ATPase?
(3) If the response to question two is positive, is this effect altered by the chronic gestational intake of L-Glu?

**Figure 3:**
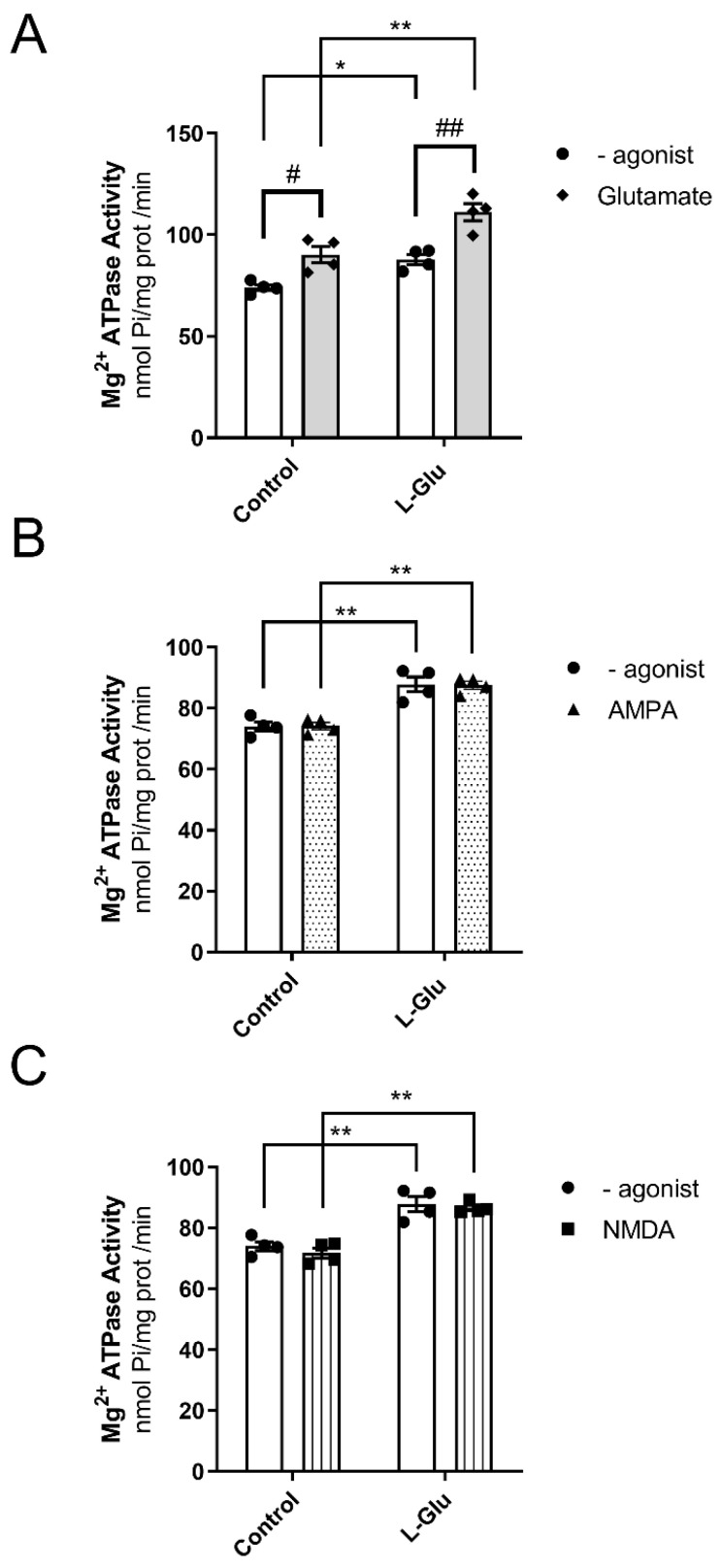
Mg^2+^ ATPase activity in the presence of Glutamate (**A**), AMPA (**B**), and NMDA (**C**) in fetal cerebellum exposed to L-Glutamate during gestation. Histograms show data that are represented as mean ± SEM of 4 different experiments performed using different plasma membrane preparations. All experiments were carried out in duplicate. * *p* < 0.05 or ** *p* < 0.01, significantly different from control values, and # *p* < 0.05 or ## *p* < 0.01, significantly different from the group without agonist, according to the two-way ANOVA test.

To answer these questions, Mg^2+^-ATPase activity was analyzed in cerebellar plasma membranes from fetuses whose mothers consumed either water or L-Glu during gestation. Furthermore, measurements were carried out in the presence of L-Glutamate, as it is an endogenous agonist of glutamate receptors. Two-way ANOVA analyses revealed the following results: (a) gestational treatment with L-Glu significantly altered Mg^2+^-ATPase activity [F(1,12) = 36.47, *p* < 0.0001]; (b) the activity in the presence of the endogenous agonist L-Glu was also significantly altered [F(1,12) = 28.74, *p* = 0.0002]; and (c) no significant interaction was found between gestational L-Glu treatment and the endogenous agonist L-Glu [F(1,12) = 1.1, *p* = 0.31]. Bonferroni’s post-test indicated that the endogenous agonist L-Glu significantly increased Mg^2+^-ATPase activity in both control fetuses (74 ± 1.5 nmol Pi/mg prot/min vs. 90 ± 4 nmol Pi/mg prot/min, *p* < 0.05) and fetuses exposed to maternal L-Glu during gestation (88 ± 2.5 nmol Pi/mg prot/min vs. 111 ± 4 nmol Pi/mg prot/min, *p* < 0.01). The post hoc test further revealed that the chronic gestational consumption of L-Glu significantly increased Mg^2+^-ATPase activity measured in both the presence (90 ± 4 nmol Pi/mg prot/min vs. 111 ± 4 nmol Pi/mg prot/min, *p* < 0.01) and absence (74 ± 1.5 nmol Pi/mg prot/min vs. 88 ± 2.5 nmol Pi/mg prot/min, *p* < 0.05) of L-Glu (Figure 3A).

Next, we wanted to identify the specific glutamate receptor that modulated the activity of Mg^2+^-ATPase. To this end, we assayed the activity of Mg^2+^-ATPase in the presence of two ionotropic glutamate receptor agonists, AMPA and NMDA. As shown in Figure 3B,C, maternal chronic L-Glu intake significantly increased Mg^2+^-ATPase activity when assayed in the absence or presence of AMPA or NMDA. However, neither AMPA nor NMDA agonists altered Mg^2+^-ATPase activity in either group of fetuses studied. Therefore, these results suggest the following: (a) the existence of a possible interaction between Mg^2+^-ATPase and the glutamate receptor other than AMPA and NMDA receptors, and (b) chronic maternal consumption of L-Glutamate throughout the gestational period increases Mg^2+^-ATPase activity.

### 3.4. Maternal Chronic L-Glu Intake Did Not Alter the Activity of Na^+^/K^+^-ATPase in Fetal Cerebellum

**Figure 4:**
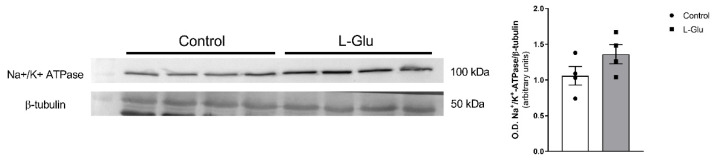
Western blot of Na^+^/K^+^ ATPase in fetal cerebellum exposed to L-Glutamate during gestation. Histograms show data that are represented as mean ± SEM of 4 different experiments performed using different plasma membrane isolations.

Next, we studied whether the level of Na^+^/K^+^-ATPase could be modulated by maternal chronic L-Glu consumption. As shown in Figure 4, the densitometric analysis of the corresponding band obtained by Western blot assays carried out with a specific antibody showed a slight, although not significant variation, in the level of Na^+^/K^+^-ATPase in cerebellar plasma membranes from fetuses exposed to maternal L-Glu.

**Figure 5:**
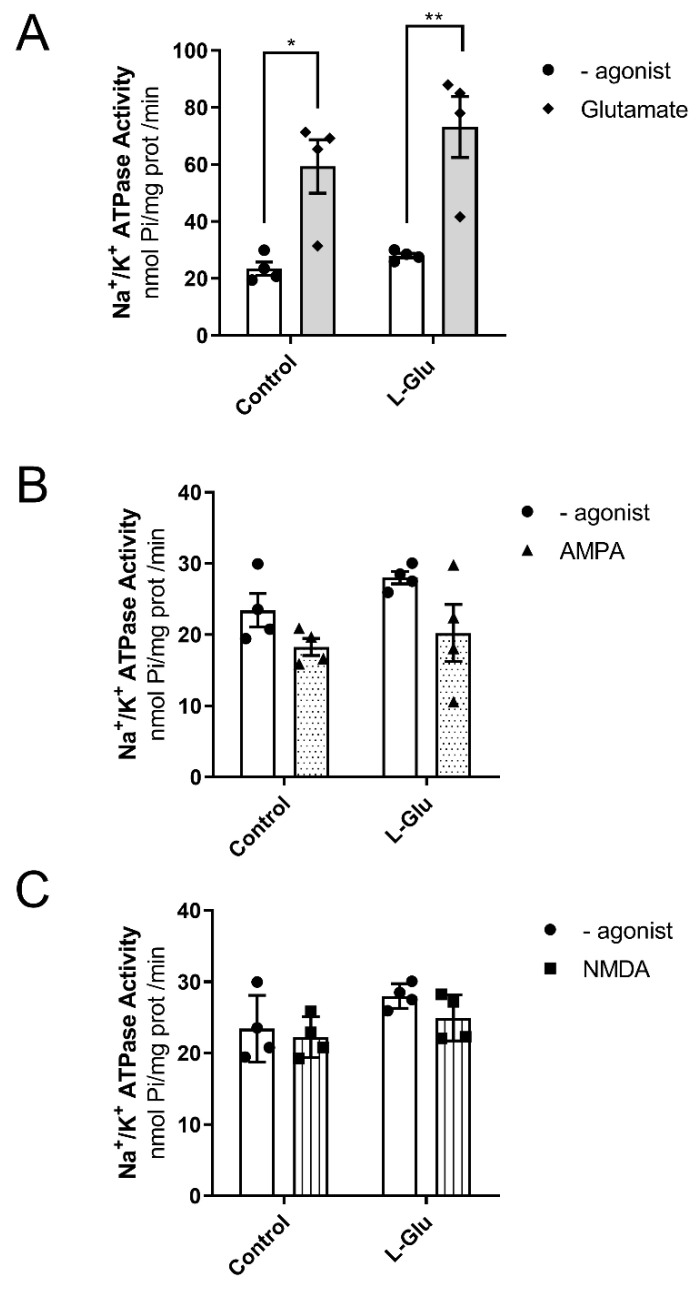
Na^+^/K^+^ ATPase activity in the presence of Glutamate (**A**), AMPA (**B**), and NMDA (**C**) in fetal cerebellum exposed to L-Glutamate during gestation. Histograms show data that are represented as mean ± SEM of 4 different experiments performed using different plasmatic membrane preparations. All experiments were carried out per duplicate. * *p* < 0.05 or ** *p* < 0.01, significantly different from control values according to the two-way ANOVA test.

We also wanted to investigate whether the activity of Na^+^/K^+^-ATPase or its coupling to glutamate receptors could also be modulated in response to the maternal intake of L-Glu during gestation. As shown in Figure 5A, the analysis of two-way ANOVA revealed the fact that chronic L-Glu consumption during gestation failed to alter Na^+^/K^+^-ATPase activity [F(1,12) = 1.69, *p* = 0.21], although the agonist L-Glu significantly affected the results [F(1,12) = 31.87, *p* = 0.0001]. Finally, no interaction between chronic treatment and L-Glu was observed [F(1,12) = 0.37, *p* = 0.55]. Bonferroni’s post-test showed that L-Glu significantly increased the activity of Na^+^/K^+^-ATPase in both control fetuses (23 ± 2.3 nmol Pi/mg prot/min vs. 59.4 ± 9.4 nmol Pi/mg prot/min, *p* < 0.05) and fetuses exposed to maternal L-Glu during gestation (28 ± 0.9 nmol Pi/mg prot/min vs. 73.2 ± 10.7 nmol Pi/mg prot/min, *p* < 0.01). Similarly to Mg^2+^-ATPase, the increase observed in Na^+^/K^+^-ATPase activity in response to glutamate receptor activation did not appear to involve either AMPA or NMDA receptors, as two-way ANOVA did not find any significant variation when these agonists were tested (Figure 5B,C).

## 4. Discussion

The present work shows how the maternal intake of L-Glu in drinking water during pregnancy produces effects on both markers related to mitochondrial dynamics and the mitochondrial electron transport chain. These effects were accompanied by changes in Mg^2+^-ATPase activity, whereas Na^+^/K^+^ ATPase activity remained unaltered.

Firstly, it was observed that treatment with L-Glu significantly decreased the levels of dynamin-related protein 1 (DRP-1) in the fetal cerebellum. This protein is a GTPase that is currently considered the main regulator of mitochondrial fission [44]. It is initially located in the cytosol, and once it is activated through GTP hydrolysis, it oligomerizes around the outer mitochondrial membrane to initiate mitochondrial fragmentation and can ultimately trigger apoptosis [45]. It is not surprising, therefore, that an abnormal increase in this process is associated with the neuronal damage observed after acute or chronic damage to the Central Nervous System [46,47,48,49,50]. On the contrary, other studies have found a reduction in the levels of this protein obtained in heterozygote knock-out animals not generating mitochondrial deficiencies; however, mice showed a reduction in oxidative stress, reducing H_2_O_2_ and lipid peroxidation levels, not affecting synaptic viability [51]. Therefore, the reduction observed in the cerebellum from fetuses could indicate a possible protective mechanism to alleviate the oxidative stress in fetuses induced by mothers exposed to L-Glu, as published in previous work [11].

Regarding the fusion proteins, OPA-1 and mitofusin 2, they play a key role in mitochondrial fusion, allowing the inner and outer mitochondrial membranes of two independent mitochondria to join and form a new single functional mitochondrion [52], which is essential for cerebellar protection against neurodegeneration [53]. However, we did not observe any significant variations in these proteins.

The change in the mitochondrial fission marker was also accompanied by a significant decrease in the levels of complex V (H^+^-transporting two-sector ATPase) and III (ubiquinone-cytochrome c reductase) detected in the group treated with L-Glu. Complex V or ATP synthase uses the energy liberated during respiration to catalyze the condensation of ADP and inorganic phosphate into ATP, providing energy to the cell [54], so a possible downregulation of this synthase could be affecting energy balance throughout the cerebellum. Also, complex III is decreased, with this being one of the main sites of ROS production in the mitochondrial electron transport chain [55], which could also be part of the same mechanism in response to augmented lipid peroxidation observed in fetuses previously [11].

ATPases are the main consumers of energy in the cell. Some studies have stated that ATP deficiency and oxidative stress may impair Na^+^/K^+^ ATPase activity and promote mechanisms of neuronal death [41]. Changes in Na^+^/K^+^ ATPase activity may have an impact on cellular metabolism and the redox state since it controls mitochondrial ROS levels and ATP utilization rates [56,57]. As changes in the levels of the mitochondrial electron transport chain were observed, these may be affecting ROS and ATP production and, therefore, ATPase functioning.

Another interesting finding from this work is the increase in the activity of Mg^2+^-ATPase in the fetal cerebellum exposed to maternal L-Glu consumption. This pump is responsible for maintaining a higher intracellular concentration of magnesium ions. This cation is essential for regulating the activities of multiple Mg^2+^-dependent enzymes and controlling the rate of protein synthesis. Additionally, it is known that magnesium plays an important role in the brain by counteracting oxidative stress, although the exact mechanism remains unclear [42,43]. Therefore, these changes suggest the existence of a protective mechanism aimed at mitigating the oxidative stress detected in the fetal cerebellum in response to maternal L-Glu intake.

The results of this study also indicate that the pump seems to be positively coupled with glutamate receptors as the activity of the pump significantly increased in the presence of the endogenous agonist L-Glutamate. However, chronic maternal L-Glu intake did not significantly alter this coupling, as it was equally augmented in the L-Glu group.

Finally, in our work, no changes were detected either in Na^+^/K^+^ ATPase activity or in the levels of the protein itself. This absence of effects contrasts with the dependence of this pump on correct mitochondrial functioning and previous works where L-Glu affects this pump’s activity through the GMP-PKG pathway [31], activating metabotropic and ionotropic receptors [30,58] and increasing Ca^2+^ intracellular levels. Although some of these experiments were performed in cerebellum slices preincubated with L-Glu at a desired concentration, and our results represent a representative dose of L-Glu orally administered through gestation, this dose is potentially too small in the cerebellum of fetuses to cause change, as the cerebellum L-Glu concentrations of these fetuses were about 4 to 5 mM of L-Glu per gram of protein, while the final glutamate concentration used for the experiments in this work was significantly higher. Also, glutamate may trigger intracellular Ca^2+^ accumulation and apoptosis, and its extrusion via the Na^+^-Ca^2+^ exchanger may be crucial for correct cell functioning, which functionally interacts with Na^+^/K^+^ ATPase activity in neurons [59]. Moreover, this pump consumes half of the ATP molecules present in the cerebellum. It is, therefore, not strange that mitochondrial alteration and the consequent production of free radicals is accompanied by a loss in the activity of this protein. Several works have shown that this pump is sensitive to oxidative stress, as a decrease in its levels has been detected in cells that have experienced oxidative damage due to hypoxia [60,61]. It has also been shown that in raw brain synaptosomes, oxidative stress significantly reduces the activity of the pump [62]. We do not know what the reasons for this may be, but they could be related to the fact that the cerebellum does not complete its development until the postnatal period, and it has been suggested that the regulation of the Na^+^/K^+^ ATPase does not conclude until development is completed [62]. Similarly to Mg^2+^-ATPase activity, the results indicate that Na^+^/K^+^-ATPase is positively coupled with glutamate receptors.

Regarding how these cerebellar alterations could affect long-term cerebellum development, we have not studied the impact of maternal L-Glutamate intake during gestation on neonatal cerebellar development. However, we believe that the significant decrease in DRP-1 levels observed in this work could be relevant, as this protein is required for cerebellar development. In this sense, the brain-specific Drp1 ablation has been shown to cause developmental defects in the cerebellum, where Purkinje cells have been shown to contain fewer giant mitochondria than controls [63]. Supporting this hypothesis, a previous study also showed that the intraperitoneal injection of monosodium glutamate (3.5 mg/g bw) for 10 consecutive days caused a significant decrease in motor coordination and the number of Purkinje cells in rats [64].

In conclusion, L-Glutamate exposure during gestation affects DRP-1 and electron transport chain complexes in the fetal cerebellum, possibly as a response to oxidative stress generated by this exposure, as reported in previous studies. The activity of Mg^2+^-ATPase was also altered by chronic maternal L-Glutamate intake, with a possible interaction observed between this activity and glutamate receptors, excluding AMPA and NMDA receptors as mediators of this effect. Finally, while the activity of Na^+^/K^+^-ATPase, one of the primary consumers of mitochondrial energy, was not altered by chronic oral L-Glutamate intake, an interaction with glutamate receptors other than AMPA and NMDA was noted.

### Limitations of the Study

This study provides indirect evidence that L-Glutamate could alter the normal functioning of mitochondria. However, more research is necessary to determine whether L-Glutamate consumption during gestation can affect mitochondrial dynamics and functioning, as well as if these possible alterations could translate into neurodevelopmental issues in offspring. In this regard, electron microscope studies could be particularly valuable, but these could not be performed in this study due to sample limitations. These will be addressed in future studies.

## Data Availability

The original contributions presented in this study are included in the article. Further inquiries can be directed to the corresponding author.
